# Pathways to professional training: a multinational study of psychotherapy trainees

**DOI:** 10.1186/s40359-026-05133-w

**Published:** 2026-08-24

**Authors:** Ulrike Willutzki, David E. Orlinsky, Armin Hartmann, Michael Helge Rønnestad, Thomas Schröder, Jan Pirke, Erkki Heinonen

**Affiliations:** 1 Department of Psychology and Psychotherapy, Witten/Herdecke University, Witten, Germany; 2 University of Chicago, Chicago, USA; 3 University of Freiburg, Freiburg, Germany; 4 University of Oslo, Oslo, Norway; 5 University of Nottingham, Nottingham, UK; 6 Finnish Institute for Health and Welfare, Helsinki, Finland

**Keywords:** Psychotherapy, Psychotherapist, Psychotherapy training, Professional development, Supervision, Wounded healer

## Abstract

**Background:**

When aspiring psychotherapists start their training they enter it with particular educational and occupational backgrounds as well as childhood family experiences. Facets of these can be regarded as patterns of pathways to psychotherapy training potentially relevant for professional development. Based on a large-scale international trainee data base and comprising a wide range of variables we analysed whether different pathway patterns could be differentiated.

**Methods:**

Pathway patterns were identified through cluster analysis using information from the *Trainee Background Information Form* of approximately 1300 trainees’ data collected in 14 countries by the SPRISTAD Collaborative Research Network*.*

**Results:**

Four pathway patterns could be differentiated defined by trainees’ educational and occupational backgrounds: Novice Psych Students; Advanced Psych Trainees; Ancillary Clinical Professionals; and Unconventional Novice Candidates. Cluster analysis also led to differentiating three pathway patterns to psychotherapy training defined by trainees’ childhood family experiences: Nurturing Families; Challenging Families; and Adversity-marked Families. Educational-occupational and childhood family pathways were generally unrelated, but each was associated with differential theoretical emphases of the training programs that trainees had selected. A comparison of pathway patterns across trainees in the five nations with most respondents showed many significant differences, especially with regard to educational-occupational pathways.

**Conclusions:**

Empirically, psychotherapy trainees entering training are not a homogeneous group but differ substantially concerning their educational/professional and family background pathway patterns to psychotherapy. The impact of these differences on training and professional development should be further explored.

**Supplementary Information:**

The online version contains supplementary material available at 10.1186/s40359-026-05133-w.

## Introduction

Psychotherapy trainings, which mostly follow general academic qualifications, are quite diverse and complex [17]. Consequently, the effects of different training elements (like work with patients, didactic elements, supervision, and self-experience or personal therapy) are difficult to disentangle. But, as current research on professional identity formation shows [24], even before being confronted with these learning areas, trainees^1^ have followed pathways into the profession that may – or may not—have an impact on how they experience and profit from psychotherapy training: Trainees have had multifaceted life and professional experiences that motivated and enabled them to embark on professional psychotherapy training. Following Kiesler (1966, 1995) generic terms like psychotherapy, psychotherapist and psychotherapy training may be regarded as ‘uniformity myths’ that obscure the delineation of potentially differentially relevant factors and their interactions in psychotherapy training and practice (see also [11]). The purpose of this paper is to examine empirically whether psychotherapy trainees beginning their training are a homogenous group and to analyze the access routes, the “pathway patterns”, to psychotherapy training. Thus, in the following, we focus on the “input”-side to training (see also [24, 27, 29]): How do trainees *enter* training, what are their educational/professional characteristics as well as their personal background, as described by themselves?

As we conceive them, *pathway patterns*^2^ to training are distinct from the individuals who follow them, and the abilities, motivations or other attributes that trainees bring with them. Pathways lead through social networks, groups, or institutions that are external to individuals who, over time, pass through them, interact with them, and are changed by them. An example of such a “standard sequence of changes” is found in the educational levels from ‘primary’ to ‘secondary’ school and, possibly, higher education. Following professional identity formation concepts [24, 27], pathways are viewed here as multifaceted routes of individuals through a social milieu, during which they develop; either by being tacitly shaped or explicitly gaining relevant skills, information, and understanding. For therapists over the life course, pathways can also be conceptualized as “primary learning arenas”, comprising “early life experience, cumulative professional experience, interaction with professional elders, and adult personal life” ([22] p. 181).

On their journey along these pathways to psychotherapy training, trainees develop characteristics that may have an impact on training outcomes. For example, in a longitudinal study over a 3-year training course, [13] found that trainees’ entry competence level determined whether there were any training effects: Trainees’ experience with the therapeutic approach (here: cognitive therapy) prior to training was positively related to competence. Moreover, only the male trainees, starting from a lower competence level compared to female trainees at the beginning of training improved over time. [26], in a trainee admission/selection group setting *before* training, looked at therapists’ interpersonal skills (as these are particularly relevant for client outcomes). They found that trainees whose interpersonal behaviors were rated more positively during the selection process had superior client outcomes in a longitudinal study over a 3-year-training course; moreover they were rated as more competent by their supervisors and had less client drop-out during training. These effects remained stable after controlling for trainee (gender, theoretical orientation, supervision etc.) as well as patient characteristics (age, gender, severity and complexity of problems). Thus, interpersonal skills vary between trainees already on entry into training (see also [15]). The examples of these studies show that ‘input’-variables to training may make a difference for psychotherapy training, and even psychotherapy process and outcome.

Pathway patterns for individuals toward psychotherapy training may be viewed as occurring in two stages. The most proximate set of pathways involves educational/occupational experiences of candidates. Educational and professional background factors may shape trainee characteristics (their overall as well as their interpersonal competence) as they start training. For example, even though they cannot be regarded as a structured training, many academic counseling or clinical programs already include courses that can be effective in fostering skills [15]; practicums in clinical settings *before* entering psychotherapy training often allow patient contact or even direct experiences in the counselor or psychotherapist role with some professional feedback. While many countries have established regulations concerning educational requirements for starting a training as a psychotherapist (see [6], online; [8]), such regulations vary markedly between countries, between professions and also between cohorts within a particular country.

Alternatively, people may already have worked in a profession not related to counseling or psychotherapy before deciding to train as a psychotherapist. Research on professional (e.g. [23]) as well as on expertise development give some hints that people who turn to a new field after already having attained proficiency somewhere else acquire skills faster, probably because they have some meta-knowledge of how to grasp essentials [5]. The impact of such differing pre-qualification patterns on developing competence in psychotherapy is largely unknown, and there is not even a description or inventory of different educational/professional pathway patterns into the profession.

Secondly, even before reaching these educational pathways, earlier pathways taken by future therapists while growing up may create motivations and capacities suited to this career (for respective reviews, see [4] and [7]). Owing to temperament and circumstances, certain childhood family pathways may lead some individuals to form an inner ‘helping’ or ‘healing’ vocation (e.g., [4, 7, 19]); others may have experienced personal therapy due to difficult family or other social contexts that convinced them to choose psychotherapy as a profession [19, 21].

While aspects of pathways into psychotherapy training are discussed in the research literature regularly [8, 24, 27], to our knowledge no empirically based description and analysis of the professional/educational and personal pathway patterns into the profession for psychotherapy trainees exists as yet. To address this gap, we used information supplied by more than 1300 trainees from 14 countries to explore how they arrived at psychotherapy training by looking for patterns of their prior educational-occupational and childhood family experiences as they experience them, taking into account a broad number of variables. Moreover, we analyzed whether the patterns of pathways were interrelated; whether these pathways influenced the type of psychotherapy training sought; and whether there were significant differences in pathways to psychotherapy in different countries.

Accordingly, the research questions investigated in this study were:

1. Does analysis of psychotherapy trainees’ educational/occupational histories reveal distinct pathway patterns followed to training? If so, what are their main characteristics?
2. Does analysis of psychotherapy trainees’ childhood family experiences reveal distinct pathway patterns followed to training? If so, what are their main characteristics?
3. What are the relationships between trainees’ educational/occupational pathways and their childhood family pathways?
4. Do educational and familial pathways that trainees have followed tend to influence the types of training they sought (e.g., their theoretical orientation)?
5. In this multi-national study, how much do the educational and familial pathways of psychotherapy trainees from different countries resemble each other?

## Methods

This study is embedded in an international longitudinal collaborative study of psychotherapy training being conducted by members of the Society for Psychotherapy Research Interest Section on Psychotherapist Development and Training (SPRISTAD; for further information on its overall design see [9, 20]).

## Design, ethics and informed consent

Since 2016, the SPRISTAD collaborative has collected information about psychotherapy training programs and trainees from different base professions and theoretical orientations. Training centers and trainees collaborating in the study have been recruited through professional publications, workshops, conferences, professional and individual collegial networks. The collaborating centers applied for ethical approvals at their local Institutional Review Boards (IRB) with material provided by the SPRISTAD collaborative (see Supplement 2 for respective ethical approvals). Trainees were included in the study if they took part in a psychotherapy training that lasted a minimum of one year (inclusion criterion); most trainees took part in psychotherapy trainings that lasted 3 + years (for a description of the psychotherapy training programs included see [17]). Data collections started when the trainees began working with patients. Trainees were informed about the study by their training institutes, signed an informed consent and provided their e-mail address to be contacted by the data collection center at Witten/Herdecke University, Germany, which sent an individualized link to fill out the forms (except in Finland, by E. Heinonen; in India by P. Bhoola and in a subset of Italian trainees by I. Messina).

## Measures

### Trainee Background Information Form (TBIF)

Data were collected with the *Trainee Background Information Form* (TBIF), a survey of trainees’ demographic, educational, professional, and childhood and current family backgrounds and experiences, as well as personal psychological characteristics (drawing on items from the *Development of Psychotherapists Common Core Questionnaire* (DPCCQ; [1, 16]). Items were either structured-response scales or checklists, with open-ended questions for textual response.^3^ The TBIF variables used to analyze pathway patterns were the following.

#### Trainees’ education and prior occupations

(a) *Highest academic degree* (5 groups): Doctorate; Master/Diplom/Magister; BA/BSc; high school/matura or less; other. (b) *Field of study* for highest degree (5 groups): Primary institutional gateways (psychology, medicine); secondary institutional gateways (related health care fields, e.g. nursing, social work, etc.); tertiary institutional gateways (education, law, social sciences); unrelated academic fields (e.g., humanistic studies, natural science, management); and Other or unknown. (c) *Previous work or professional experience* (7 groups): Primary mental health professions (psychology, psychiatry); ancillary health professions (e.g., clinical social work, psychiatric nursing, art/dance/music therapies); peripheral academic fields (education, law, social sciences); remote academic fields (humanistic, natural science); non-academic fields (businesses, service industries, sports); university studies (field unknown); none reported. (d) *Therapeutic relevance of prior work* (6 groups): Directly relevant (psychological treatment); indirectly relevant (other clinical mental health settings); irrelevant for psychotherapy (e.g., business, sports, military service); work claimed but unspecified; none; unclear. (e) *Amount of prior psychotherapeutic work* (3 groups): no prior practice; up to 4 years; more than 4 to 10 years; 10 to 30 years). (f) Experience of prior *supervision* (2 groups): none, some.

#### Trainees’ childhood family backgrounds and experiences

Trainees were asked about their childhood family backgrounds and experiences: (a) Childhood family’s *religious tradition*, (coded in 4 groups): Roman Catholic; Protestant or ‘Christian’; other religious traditions, e.g., Jewish, Muslim, Hindu; no religion; not answered. (b) Childhood family’s status as *socio-ethnic minority* or *mainstream majority* (coded as no/yes). (c) Childhood family’s *economic status* (4 groups): Very comfortable; comfortable; sufficient; insecure. (d) Childhood family’s size (5 groups): One child, 2 child family; 3 child family; 4 + child family; no information). (e) Trainee’s *birth-order* (5 groups): Only child; oldest child; middle child; youngest child; no information. (f) *Divorce or separation* of trainee’s parents (no/yes). (g) Trainees’ rating of childhood family’s *emotional/psychological functioning* (6-point scale, 3 groups: 4–5 Good/excellent; 3 moderate; 0–2 poor). (h) Trainees’ rating of *care/support* experienced growing up (6-point scale, 3 groups: 4–5 loved; 3 maintained; 0–2 neglected). (i) Trainees’ rating of *abuse/trauma* experienced growing up (6-point scale, 3 groups: 4–5 harmed; 2–3 distressed; 0–1 protected). (j) Trainees’ having experienced *personal psychotherapy*, other than required for training (yes/no). (k) Trainees’ rating of how much personal issues/problems influenced their motivation to seek training as psychotherapist (6-point scale, 2 groups: 3–5 moderately or more; 0–2 some or less).

#### Other variables

For sample description, the following variables were included: (a) Trainees’ age; gender; marital/relationship, parental status; (b) Training program’s nation, duration, theoretical orientation. (c) Trainees’ views of *program’s theoretical emphases* (six 6-point scales, 0 = not at all, 5 = very greatly), with trainees rating all scales (analytic/psychodynamic; behavioral; cognitive; humanistic; interpersonal; systemic) and ratings of 4 (greatly) or 5 termed ‘salient’.

### Sample

The sample comprised 1,345 psychotherapist trainees (see Table 1). Average age was 35 years, with 72.1% of trainees under 40. 84% of the trainees were female. 14 countries were represented, with approximately three quarters residing in five European countries (Austria, Finland, Germany, Italy, UK). Personally, 58.7% of the trainees were in a committed relationship and more than a third had children. In terms of academic background, 60.7% had earned a higher university degree, 20.2% a bachelor’s degree, while a small group (6.5%) had only high school education or less.

**Table 1:** Trainee sample: individual and program characteristics

Age	*M/Med*	*SD/Range*
Years	35.3/32.6	9.6/19–70
Age Groups	*n*	%
19–29	513	38.4
30–39	450	33.7
40–70	373	27.9
Sex	*n*	%
Female	1135	84.4
Male	210	15.6
Marital Status	*n*	%
Single	497	38.3
Living w. partner	336	25.9
Married	425	32.8
Separated/Divorced	38	2.9
Parental Status	*n*	%
Has children	470	35.0
Academic Preparation	*n*	%
Doctoral	82	6.1
Masters/equivalent	736	54.6
Bachelor	272	20.2
High school or less	86	6.40
Not reported	173	12.8

Prior Practice	*M/Med*	*SD/Range*
Years	2.0/0.0	4.5/0–29
by Group	*n*	%
0.0 years	945	76.0
> 0–0—4 years	120	9.7
> –4—10 years	103	8.3
≥ 10 years	75	6.0
Program Location	*n*	%
Argentina	47	3.5
Austria	276	20.5
Canada	11	1.9
Chile	59	4.4
Finland	252	18.7
Germany	137	10.2
India	94	7.0
Ireland	4	0.3
Italy	235	17.4
Lithuania	31	2.3
Romania	12	0.9
Switzerland	44	3.3
United Kingdom	132	9.8
United States	15	1.1

Program Duration	*M/Med*	*SD/Range*
Years	*3.7/4.0*	*1.1/9*
By Group	*n*	%
≤ 3 years	299	27.5
> 3 & ≤ 4 years	565	52.0
> 4 & ≤ 6 years	223	20.5
Program Orientation^1^	*n*	%
Psychodynamic	453	36.4
Cognitive-Behavioral	327	26.3
Humanistic	414	33.3
Interpersonal	427	34.3
Systemic	271	21.8
Personal Therapy^2^	929	69.2

Not: *N* ≅ 1345

^1^‘Salient’ (great or very great) theoretical program emphasis; multiple emphases allowed

^2^Other than as training requirement

By far most of the trainees were novices, without prior experience as psychotherapists when starting the psychotherapy training evaluated in the longitudinal SPRISTAD-study. However, about one-fifth were more advanced in their training, with about one in seven reporting over 4 years in therapeutic practice. Most training programs required 4 years duration, with approximately three quarters lasting from 3 up to 6 years (inclusion requirement: minimum program duration of 1 year). All major therapy orientations were represented (trainees could rate each orientation, hence adding to more than 100%). 36.4% of the programs were rated as saliently analytic/psychodynamic, 26.3% interpersonal, 33.3% humanistic, 26.3% as saliently cognitive-behavioral, and 21.3% as saliently systemic. 69.2% of the trainees had experienced psychotherapy themselves independently of training requirements.

### Statistical methods

Statistical analyses were performed with SAS-JMP (v13) and SPSS (v28). For sample description, we computed means, medians, SD, range, frequencies and percentages. Differences were tested by ANOVAs, including Scheffé tests for contrasts. Interrelationships between variables were tested by correlation coefficients or contingency tables. To identify significant deviations of cell frequencies from expected values, we used adjusted residual values (AR) with the critical AR threshold in a footnote to the relevant tables. Only ARs with a Bonferroni corrected significance level (divided by number of table cells) of *p* = 0.05 were interpreted.

Cluster Analyses [3] were conducted separately for educational/occupational and childhood family variables to establish groups of similar trainees. To use the platform Latent Class Analysis (SAS-JMP online) for cluster analyses for nominal variables, we categorized numeric variables into subgroups by dividing the numeric variable’s range. Each level of an unobservable (i.e., inferred) variable is defined as latent class. The platform fits a latent class model and assigns each observation to its most likely class (cluster). It provides BIC and AIC statistics to determine the optimal number of clusters in terms of best fit. Typically, BIC and AIC point to different optimal solutions (e.g., optimal number of clusters). In this range, we choose the most parsimonious model with the best face validity. We also report classification certainty (range 0–1; higher values indicating better classification) and relative entropy (following the Mplus convention: range 0–1, higher values indicating better classification) for all cluster solutions presented. As Sorgente et al. [28] acknowledge, no agreed-upon thresholds currently exist in the field. That said, values above 0.80 are widely cited as acceptable for both classification certainty and relative entropy (Sorgente et al., 2025). With respect to the lower bound, Weller et al. [30] note that an entropy value below 0.60 would likely preclude publication.

The Latent Class Analysis shows derived clusters in columns and defining variables in rows, subdivided by categories of each variable. Each cell of that table contains the conditional probability of the response indicated by row. Imagine a dichotomous variable (e.g., ‘having supervision’ Y/N) with a base rate of Y = 47% for the whole sample. In the respective cell of a cluster one might find the conditional probability of cp = 13%. This value indicates that trainees in this cluster were less likely to have had supervision.

As the strength of a cellwise deviation is hard to evaluate (since it depends on base rates and direction of deviation), we devised base rate categories as follows: The conditional probabilities deviate from each base rate to either 0% or 100%. The room for deviation is equal for a base rate of 50% (max. deviation = 50%), but for a base rate of 20% max. deviations are 20% (towards 0%) and 80% (towards 100%). Simply calculating the difference between base rate and cell percentage is potentially misleading. With a base rate of 20%, a deviation of 10% is close to 0%, but a long way from 100%. To account for this imbalance, a classification of weighted deviations into five categories (–,-,o, +, + +) is provided. A weighted deviation for each direction of deviation (wd = (base rate – cp)/avail variation) was computed. In case of a base rate of 13%, for deviations with cp < 13% the weight is 13; for deviations with cp > 13% the weight is 87. The resulting weighted deviations range from −100% to + 100%. The cut-off for the five categories are (–60%,—20%, + 20%, + + 60%). Strength of the relationship of single variables with the whole cluster was determined via Cramer’s V, interpretable like a correlation coefficient: small 0.1 to 0.3; medium 0.3 to 0.5; strong > 0.5 [2].

In considering which variables to use for differentiating pathways, the following variables were not retained in the Cluster Analysis: (a) Trainee’s family’s religious tradition was reported by few and the remainder (*n* = 721) skewed largely to Christian denominations; (b) minority status (social, cultural or personal) was rare (8%); (c) family size and trainee birth order were strongly overlapping and both not contributing to differentiating clusters; (d) parental divorce or separation was not uncommon (31% of trainees), but covaried with family function and family economic status.

## Results

### Educational and occupational pathways

The cluster analysis of trainees’ educational and occupational characteristics yielded four distinct pathway patterns to psychotherapy training, together accounting for 1125 trainees (see Table 2).

**Table 2:** Educational-occupational clusters: percentages and base-rate deviations

Variable	Value	Educational-occupational pathways									
		Novice psych students		Advanced psych trainees		Ancillary clinical professions		Unconventional novice candidates		Base %^1^	Cramer’s V^2^
Highest academic degree	Doctoral	7%	o	10%	o	0%	–	1%	–	6%	.55
	Masters	70%	+	71%	+	41%	-	0%	–	59%	
	Bachelor	23%	o	15%	-	51%	+	4%	–	23%	
	Secondary or less	0%	–	0%	–	0%	–	67%	+ +	7%	
	Other	0%	–	4%	o	8%	o	27%	+	5%	
Academic degree field (1st listed)	Psychol/medicine	74%	+	96%	+ +	0%	–	9%	–	62%	.63
	Ancillary clinical	2%	–	0%	–	85%	+ +	6%	-	13%	
	Educ. & SocSci	8%	o	1%	–	11%	o	12%	o	7%	
	Unrelated fields	16%	o	3%	–	3%	–	32%	+	13%	
	Other/unknown	0%	–	0%	–	1%	–	41%	+	5%	
Previous profession or training	Psychol, psychiat	6%	-	34%	+	3%	–	2%	–	11%	.54
	Ancillary clinical	3%	–	4%	–	57%	+	5%	-	10%	
	Educ/Soc. Science	11%	o	4%	-	4%	-	17%	o	9%	
	Hum/Nat. Science	8%	o	3%	-	0%	–	2%	–	5%	
	Business/Other	23%	o	4%	–	7%	–	41%	+	19%	
	U. student/unspec	49%	o	49%	o	30%	-	0%	–	42%	
	None reported	0%	–	2%	-	0%	–	34%	+	4%	
Relevance of prior training and work	Direct: Tx-related	3%	–	40%	+	29%	o	0%	–	14%	.32
	Indirect: Clinical	24%	o	31%	o	39%	o	17%	-	27%	
	Irrelevant: Other	20%	o	5%	–	6%	–	33%	o	16%	
	Claimed, unlisted	4%	o	1%	–	0%	–	0%	–	2%	
	None	44%	o	14%	–	25%	-	44%	o	35%	
	Unclear	5%	o	10%	o	2%	–	5%	o	6%	
Prior therapeutic practice	0 years	100%	+ +	11%	–	48%	-	96%	+ +	75%	.74
	> 0 to 4 years	0%	–	39%	+	14%	o	1%	–	10%	
	> 4 to 10 years	0%	–	29%	+	22%	o	0%	–	9%	
	> 10 to 30 years	0%	–	21%	o	16%	o	3%	-	7%	
Prior supervision	Yes	13%	-	98%	+ +	82%	+ +	23%	-	41%	.49
	No	87%	+ +	3%	–	18%	–	77%	+	60%	
	n	646		222		138		119		1125	

Min BIC = 14 405.6; Classification Certainty = 0.95; Relative Entropy = 0.93

^1^Base-rate deviation = distance of observed % from base % by extent of available variation: + + much higher; + higher; 0 neutral;—lower; – much lower

^2^Cramer's V Interpretation: small 0.1 to 0.3; medium 0.3 to 0.5; strong > 0.5

The first pathway pattern, accounting for more than half the sample, was identified by consisting of students in Psychology or Medicine having no previous experience in psychotherapy. Most had attained graduate level education and none had less than a bachelor degree. For half of these novice trainees, university study was their only ‘occupation’. We called this main traditional academic pathway to psychotherapy training *Novice Psych. Students* (‘Psych’ to include Psychology, Psychiatry or Psychosomatics). Trainees on the second pathway pattern were very similar to *Novice Psych. Students* regarding traditional academic routes —except for having already been in training and consequently being clinically more experienced. We called those on this pathway *Advanced Psych. Trainees*, as 89% had already done some psychotherapy and 98% had been in supervision. The remaining two pathway patterns identified by the cluster analysis represent variant routes to psychotherapy training, taken by nearly a fourth of our sample. Pathway pattern three, the *Ancillary Clinical Professions,* consisted of persons with prior training/occupational experience in mental health related areas, including psychiatric nurses, clinical social workers, and other professionals commonly working in mental health settings (e.g., art, dance, music, occupational, and physical therapists). More than half had participated in therapeutic work (e.g., as co-therapists in group, ward or family treatments), and most had been supervised. Even more distinct from the traditional pathways to psychotherapy training was a largely unconventional, nonacademic route, followed mostly by persons having only secondary (high school/matura) level of education, or less, that we called *Unconventional Novice Candidates*. Their occupations were very heterogenous, such as business, finance, banking, diplomatic service, police or military service, tourism, massage, music, design, publishing, preschool teaching, etc. Virtually none of them had previous experience doing therapy; 70% had already been in personal therapy, which is comparable to those on other pathways. The variables most strongly differentiating the educational/occupational pathway patterns were: Prior Therapeutic Practice (V = 0.74); Academic degree field (V = 0.63); Highest academic degree (V = 0.55); and Previous profession/training (V = 0.54).

### Childhood family pathways

Cluster analysis of the variables describing trainees’ childhood family backgrounds/experiences revealed three different pathway patterns to psychotherapy training (Table 3).

**Table 3:** Childhood family clusters: percentages and base-rate deviations

Variable	Value	Childhood family pathways							
		Nurturing families		Challenging families		Adversity-marked families		Base % ^1^	Cramer’s V ^2^
Childhood family economic situation	Luxury	21%	o	12%	o	7%	-	14%	.21
	Comfortable	50%	o	43%	o	32%	-	43%	
	Just Sufficient	20%	-	34%	o	36%	o	28%	
	Insecure/Marginal	10%	-	12%	o	25%	o	15%	
Family psych & emotional functioning	Poor/Lacking	**1%**	–	18%	-	92%	+ +	31%	.71
	Adequate	20%	-	69%	+	**7%**	–	31%	
	Good	79%	+ +	13%	–	**1%**	–	38%	
Experienced care & support growing up	Felt Ignored	**0%**	–	0%	–	43%	+	12%	.57
	Felt Maintained	**1%**	–	26%	o	42%	+	20%	
	Felt Loved	99%	+ +	74%	o	15%	–	68%	
Experienced trauma or abuse growing up	Felt Protected	93%	+ +	59%	o	30%	-	66%	.40
	Felt Distressed	**5%**	–	28%	o	37%	+	21%	
	Felt Harmed	**2%**	–	12%	o	33%	+	14%	
Personal therapy (not for training)	Yes	51%	-	89%	+	88%	+	72%	.29
	No	49%	+	11%	-	12%	-	28%	
Personally motivated career choice	Little or None	60%	+	29%	-	28%	-	42%	.23
	Somewhat	30%	-	47%	o	40%	o	37%	
	Strongly	11%	-	24%	o	32%	o	21%	
	n	532		342		340		1214	
	%	43.8%		28.2%		28.0%		100%	

Min BIC = 11 651.1; Classification Certainty = 0.79; Relative Entropy = 0.69

^1^Base-rate deviation = distance of observed % from base % by extent of available variation: + + much higher; + higher; 0 neutral;—lower; – much lower

^2^Cramer's V interpretation: small 0.1 to 0.3; medium 0.3 to 0.5; strong > 0.5

The first cluster (44% (*n* = 532) of the sample) was characterized mainly by highly positive ratings of the families they grew up in (i.e., as functioning very well, emotionally/psychologically). Almost all felt they had been well protected from trauma/abuse. About two-thirds (61%) were born into economically well-off families. The fortunate trainees on this happy pathway clearly felt that their early pathway led through basically *Nurturing Families*. About half (49%) seemingly never felt a need to have therapy for themselves, and 60% indicated that personal issues or problems did *not* motivate them to become psychotherapists. In the second cluster (28% (*n* = 342) of the sample) 69% of trainees experienced their childhood family’s emotional/psychological functioning as merely adequate. Nevertheless, most (74%) felt loved/protected, although 26% felt they were just maintained rather than loved, and as many as 40% felt distressed/harmed during childhood. Material conditions while growing up were also not experienced as benign for trainees whose path to training started from more difficult or *Challenging Families*. Almost all (89%) had sought therapy independently of training requirements, and a clear majority (71%) reported that personal issues/problems had at least somewhat or even strongly influenced their wish to become professional therapists. The trainees in the third cluster (28% (*n* = 340) of the sample) almost all (92%) rated their childhood families as functioning poorly in emotional/psychological terms. Less than 15% had felt loved; 42% felt at least having been ‘maintained’ (e.g., fed and clothed, if not loved), but a similiarly large group (43%) felt they had been neglected. Likewise, only 30% of these trainees reported to have been protected as children, while 70% had either experienced significant emotional distress or reported they had been harmed by trauma/abuse. We called this pathway pattern *Adversity-marked Families*— being adversity-marked at least in regard to providing a secure context for children. Unsurprisingly, 88% of these trainees had already sought personal therapy, and 72% said that their own personal issues/problems had motivated them, either somewhat or strongly, to become a psychotherapist. The variables most strongly differentiating the childhood family pathway patterns were: Family emotional/psychological functioning (V = 0.71); Experienced care/support growing up (V = 0.57); and Experienced trauma/abuse (V = 0.40).

### Relation of educational/occupational and childhood family pathways

The third research question concerned significant relations between educational/occupational pathway and childhood family pathway patterns. Although the overall probability of the cross-tabulation of Educational/Occupuational by Family Pathway pattern was significant (*χ*^2^ (6) = 15.5, *p* = 0.02), none of the individual cell values reached a significant level (see Supplement Table A1). Thus, it appears that childhood family and educational/occupational pathway patterns are relatively independent.

### Relation of educational-occupational and childhood family pathways to training program orientations

Concerning relations between the pathway patterns to training and theoretical orientations of training programs, differences in training programs’s theoretical orientation were found for all scales except for that of Cognitive-behavioral (see Table 4).

**Table 4:** Training program orientation by family and educational pathways*

*From* … training pathways	*… To*: training program theoretical emphasis				
	Analytic/dynamic	Cognitive-behavioral	Humanistic emphasis	Interpersonal emphasis	Systemic emphasis
Education-occupation	F = 5.88, *p* <.001	F = 0.76, *p* = ns	F = 29.7, *p* <.001	F = 8.38, *p* <.001	F = 11.1, *p* <.001
Novice Psych. Students	5.01^a^ (.15)	4.70 (.15)	3.94^a^ (.12)	5.77^b^ (.12)	4.78^b^ (.10)
Advanced Psych. Trainees	5.32^a, b^ (.25)	4.77 (.22)	5.65^b^ (.21)	4.88^a, b^ (.21)	3.80^a^ (.13)
Ancillary Clinical Professions	4.46^a^ (.30)	4.87 (.29)	5.39^b^ (.28)	4.53^a^ (.27)	3.56^a^ (.27)
Unconventional Novice Candidates	6.29^b^ (.30)	5.17 (.25)	7.03^c^ (.24)	5.39^a, b^ (.29)	5.20^b^ ()

Childhood Family	F = 7.98, *p* <.001	F = 24.4, *p* <.001	F = 8.71, *p* =.001	F = 3.22, *p* =.04	F = 3.16, *p* =.04
Nurturing Families	4.68^a^ (.16)	5.58^b^ (.14)	5.24^a^ (.13)	5.35^a, b^ (.13)	4.00^a^ (.13)
Challenging Families	5.54^b^ (.21)	4.52^a^ (.18)	5.24^a^ (.19)	5.13^a^ (.19)	4.29^a^ (.18)
Adversity-marked Families	5.35^b^ (.20)	4.04^a^ (.09)	6.07^b^ (.18)	5.75^b^ (.18)	4.32^a^ (.18)

^*1^way ANOVAS with Scheffe test: cell numbers indicate subgroup *M*s & (SE); different letters indicate significantly different subgroups

For *Educational-Occupational* pathway patterns and theoretical orientations: (a) *Novice Psych Students* were significantly higher statistically on the Interpersonal, and lower on both Analytic/psychodynamic and Humanistic; (b) *Advanced Psych Trainees* were higher on Humanistic and lower on Systemic; (c) *Ancillary Clinical Professionals* were higher on Humanistic and lower on Analytic/psychodynamic, Interpersonal and Systemic; (d) *Unconventional Novice Candidates* were higher on Humanistic, Analytic/dynamic, and Systemic, and bridged higher and lower groups on Interpersonal.

For *Childhood Family* pathway patterns, statistically significant differences in program orientation were found for all theoretical orientations: (a) programs chosen by *Nurturing Family* trainees were higher on Cognitive-Behavioral program emphases, and lower on all others; (b) programs chosen by trainees from *Challenging Family* had significantly higher Analytic/psychodynamic orientation and lower on others; (c) programs chosen by trainees from *Adversity-marked Families* were higher on Humanistic, Interpersonal, and Analytic/dynamic orientations, and lower on Cognitive-Behavioral.

### Educational-occupational and childhood family pathways in different nations

Finally, the fifth research question asked whether educational-occupational and childhood family pathway patterns to psychotherapy training are similar or different among trainees in the main five countries (Austria, Finland, Germany, Italy, UK) in our sample (Table 5).

**Table 5:** Educational-occupational and family pathways by countries

Pathways		Countries					Total
		Austria	Finland	Germany	Italy	UK	Total
Educational-Occupational							
Novice Psych Students	n	97	39	96	196	102	530
	%	**18.3%**	**7.4%**	**18.1%**	**37.0%**	**19.2%**	100.0%
	AR^1^	−5.9	−14.0	5.4	11.6	5.4	
Advanced Psych Trainees	n	6	91	19	15	6	137
	%	**4.4%**	**66.4%**	13.9%	**10.9%**	**4.4%**	100.0%
	AR^1^	−6.2	12.5	.3	−3.7	−3.5	
Ancillary Clinical Professions	n	36	89	3	1	8	137
	%	26.3%	**65.0%**	**2.2%**	**0.7%**	5.8%	100.0%
	AR^1^	.2	12.0	−4.1	−6.8	−3.0	
Unconventional Novice Candidates	*n*	96	4	1	1	12	114
	%	**84.2%**	**3.5%**	**0.9%**	**0.9%**	10.5%	100.0%
	AR^1^	15.3	−5.5	−4.1	−6.0	−1.1	
	*n*	235	223	119	213	128	918
	%	25.6%	24.3%	13.0%	23.2%	13.9%	100.0%
Pearson χ^2^ = 634.0 df=12 *p* <.001				df = 12		p	
Childhood Family							
Nurturing Families	*n*	134	122	90	107	25	478
	%	28.0%	25.5%	**18.8%**	22.4%	**5.2%**	100.0%
	AR^1^	.4	1.6	4.5	-.3	−6.8	
Challenging Families	*n*	70	56	29	43	32	230
	%	30.4%	24.3%	12.6%	18.7%	13.9%	100.0%
	AR^1^	1.2	.4	-.5	−1.7	.6	
Adversity-marked Families	*n*	71	56	18	79	71	295
	%	24.1%	19.0%	**6.1%**	26.8%	**24.1%**	100.0%
	AR^1^	−1.5	−2.1	−4.5	1.9	6.9	
	*n*	275	234	137	229	128	1003
Pearson χ^2^ = 82.2 df=8 *p* <.001				df = 8		p	

^*^Cell percentages in bold type diverge from expected significantly at *p* ≤.01

^1^*AR* Adjusted standardized residual

For Educational-Occupational pathway patterns, the $\chi^2$ analysis, with percentages calculated for pathways, show many significant differences: (a) *Novice Psych Students* were largely from Italy, UK and Germany, whereas Austria and especially Finland had fewer than expected; (b) *Advanced Psych Trainees* were mostly from Finland, while other countries had fewer than expected; (c) *Ancillary Professional Candidates* were also mainly from Finland, while Germany, UK and especially Italy had fewer than expected; (d) *Unconventional Novice Candidates* were almost all from Austria; very few from other countries.

By contrast, few significant results were found for Childhood Family pathway patterns: (a) *Nurturing Family* trainees came proportionally more (although numerically less) often from Germany, and from the UK both proportionally and numerically less often; (b) *Challenging Family* trainees did not deviate significantly from expectations; (c) *Adversity-marked Family* trainees were proportionally more often from UK, and proportionally less from Germany.

Combining educational-occupational and childhood families pathway patterns, one observes: (a) *Austrian* trainees were mostly either Novice Psych. Students or Unconventional Novice Candidates, with a statistically expected distribution over Childhood Family pathway patterns; (b) *Finnish* trainees were mostly either Advanced Psych. Trainees or Ancillary Clinical Professionals, with again the distribution of Childhood Family pathway patterns as expected; (c) *German* trainees were most commonly Novice Psych. Students, and more than expected by chance from Nurturing (66%) and less from Adversity-marked Families; (d) *Italian* trainees were almost all Novice Psych Students, and as expected from Childhood Family pathway patterns; (e) *United Kingdom* trainees, like those from other countries, were mostly Novice Psych Students, but in contrast to other countries, an unexpectedly large percentage had been on an Adversity-marked Family pathway pattern, with correspondingly fewer than expected from Nurturing Families.

## Discussion

In sum, in this exploratory study distinct patterns of pathways to psychotherapy training were found with cluster analyses of variables describing trainees’ educational/occupational attainments and, separately, through cluster analysis of variables describing trainees’ memories and rating of their childhood family experiences. The classification of educational pathways meets statistical criteria and appears substantively valid. The classification of family backgrounds is statistically weaker, yet yields clearly interpretable classes. The comparatively lower classification quality in the latter case may partly reflect the cultural heterogeneity inherent in our multinational sample, which could introduce additional within-class variation that the model cannot fully absorb. These two sets of pathway patterns were found to be largely independent, although the most common combination led from Nurturing Families to Novice Psych Students. The different educational/occupational and childhood family pathway patterns to training taken by individuals were both significantly related to the theoretical emphases of the programs chosen. Finally, the pathway patterns to psychotherapy training – especially the educational/occupational pathways – varied considerably between nations. As the cluster analyses led to statistical groupings of trainees’ reports about their education and work as well as their perceived family experience prior to beginning their psychotherapy training they are not longitudinal even though the information provided refers to different points in time. Moreover, as all information is self-report, it has to be kept in mind that we are not dealing with descriptions of facts but with current reconstructions of both educational/occupations and family experiences. With these caveats in mind, we allow us to hypothesize about potential benefits as well as drawbacks of such pathway patterns on trainees’ therapeutic practice as well as their further professional development, taking into account relevant research and literature.

## Pathways

### Educational-occupational pathways

Four educational-occupational pathway patterns were distinguished, but inspection of their content suggests that two are likely the same pathway viewed at different points in trainees’ life course. Those on the *Novice Psych Student* pathway had no previous psychotherapy practice and are just at the point of entering training; most on the *Advanced Psych Trainee* pathway resemble *Novice Psych Students* (e.g., as having advanced degrees in psychology or medicine), except that they were already involved in supervised therapeutic work, had been in practice before and were taking an additional course of training. These psychological/medical academic pathway patterns to psychotherapy training, followed by 77% of the sample, clearly represent the primary route of trainees up to initial, as well as further training.

The other two educational-occupational pathway patterns represent clearly minor routes to psychotherapy training, respectively covering 12% and 11% of the total sample. The first of these pathways, *Ancillary Clinical Professionals*, involves trainees with prior clinical experience in mental health work, either directly or indirectly related to psychotherapy. They have undergraduate or graduate-level university backgrounds. Many had already participated in some forms of therapeutic activity and supervision. If one views being a fully trained psychotherapist as a higher vocational status, these trainees can be understood as upgrading established professional competences.

The fourth educational-occupational pathway pattern is the most unusual and probably least recognized route by which people become engaged in psychotherapy training. Those *Unconventional Novice Candidates* did not have the academic training, but in fact, educationally, most had neither a graduate nor an undergraduate degree. Neither did their prior work experience involve the mental health field. It appears that their main prior involvement was as patients having their own therapy, which may have become a life-changing inspiration, redirecting their pursuits and commitments. On the one hand, the respective countries’ regulations must allow them to train as psychotherapists; on the other hand these trainees may possess some unusual personal qualities and talents that made them seem suited for therapeutic work. While academic qualifications are in most countries academic qualifications considered an advisable or necessary preparation for psychotherapeutic work [8] the notion that they are necessary for or at least foster professional development and competence has also been challenged (for example see [14]).

### Childhood family pathways

While the Educational/Occupational variables may seem ‘sociological’ in content, the variables used in defining Childhood Family pathway patterns were distinctly psychological, i.e., trainees’ *recollections and evaluations* (not ‘objective’ conditions that observers might have noted about those families decades earlier) of the families they grew up in. Our findings may help to resolve a debate in the literature on experiences of adversity in therapists’ childhood families, where results both supported and rejected childhood adversity as a source of therapists’ career choices and suitability [4, 7]. The present study shows that differences of opinion are most likely due to there being three Childhood Family pathway patterns. Almost half of the trainees in our sample started their journey to training from well-functioning *Nurturing Families*, whereas many other trainees started from either *Challenging* or *Adversity-marked* Families.

#### Nurturing families

The largest group of trainees (46%) were on a pathway leading from Nurturing Families to psychotherapy training, with families that functioned well. Almost all of them felt that their choice of profession was not at all or at most somewhat related to their own need for healing; and unlike other trainee clusters, only half had been in therapy. These trainees seem likely to become the professional therapists described as Normal Caring Healers in the study of more than 10,000 psychotherapists conducted by the SPR Collaborative Research Network (SPR/CRN) [16, 18]. Very likely they are the same referred to as “healing healers” [sic] in a recent research review by [4]. Based on their life histories and individual qualities, Normal Caring Healers at best “…draw on their own extensive experience as participants in and beneficiaries of past and present caring relationships that foster personal development; which in turn, builds a deep well of warmth and self-bestowal toward cared-for others; and which creates an infectious sense of good cheer or positive morale they can offer to the typically ‘demoralized’ patients who come for therapy” [19] p.186; see also [7]. On the other hand, there is a risk that excessive nurturing might foster something like the “healthy-mindedness”, classically described by William [12]: A consistent cheerfulness, optimism, and tendency to view problems as solvable might develop among such trainees, and this may lend itself to a superficial view of suffering which may not feel empathetic to patients struggling with what seem unresolvable conflicts.

#### Challenging families

Trainees from Challenging Families more often described an adverse early environment. Over two thirds evaluated their family’s functioning as just adequate, with another fifth rating it as poor. While a majority of trainees had felt loved and protected from trauma/abuse, two out of five had felt distressed or harmed while growing up, and a quarter felt they had been just maintained rather than loved. Nine out of ten had sought personal therapy independently of training requirements, and over two thirds judged that own issues and problems at least somewhat motivated them to become psychotherapists. It is hard to guess what trainees starting from Challenging Families might come to be like as professional practitioners. The majority who had felt loved and protected while growing up might find their way to becoming Normal Caring Healers. The eventual outcome of those who had felt only maintained and exposed to distressing or harmful levels of trauma in childhood might depend on their personal resilience and the healing experienced in their personal therapy. At the same time, they might become better able to draw on their own experiences of overcoming adversities and resolving personal difficulties to empathize with and understand how to help patients. Having worked successfully on their own healing might strengthen them into becoming Wounded Healers [4, 7, 16].

#### Adversity-marked families

Least well-off are the 28% of trainees in our sample who began their journey as therapists in Adversity-marked Families. Virtually all evaluated their family’s emotional/psychological functioning as poor and said they were either merely maintained or outright neglected. Perhaps even worse, fewer than a third felt they had been protected. Again, these circumstances may have either a favorable or problematic impact on trainees’ professional development. With sufficient work on personal difficulties, some may become truly helpful as the Wounded Healers that were described in the SPR/CRN study: “… healers who became such after they themselves were healed sufficiently from their own wounds …[who**]** engaged extensively in their own personal therapy, benefitted substantially from it, and also attained the highest levels of Effective Practice” ([19] p. 184–5). On the other hand, the pathway through Adversity-marked families may produce potentially serious psychological casualties when being burdened by patients’ hardships. Some trainees may become Indifferent Healers (as described above) or, like those in the SPR/CRN study, appear to be Troubled Healers who “…experience therapeutic work as a Distressing Practice, an ordeal that was difficult and disturbing for them” ([19] p. 188). Nevertheless, it is important to remember that trainees from Adversity-marked Families have become functional adults with sufficient abilities to have been accepted into a professional psychotherapy training program. In most countries, this takes both sufficient intellectual as well as personal qualities (e.g., good academic grades, empathy, self-awareness, and mental health; [17]).

### Pathways correlates

#### Correlations of educational-occupational and childhood family pathway

The last point is reinforced by the fact that no statistically significant links between Childhood Family and Educational-Occupational pathways were found. Essentially, trainees who experienced one or another kind of family while growing up were about equally likely to enter psychotherapy training by any of the educational-occupational pathways. Nevertheless it is worth remembering that the largest group of trainees in our sample came from Nurturing Families to become *Novice Psych Students* (followed by *Advanced Psych Trainees*).

### Program correlates

Pathway patterns predicted at least one important aspect of the programs that trainees had selected—their main theoretical emphases that is related to a particular view on human being and growth.

#### Educational-occupational pathway program correlates

*Unconventional Novice Candidates* had chosen programs that they saw as significantly more Analytic/Psychodynamic, and more Humanistic and Systemic in orientation. One hypothesis would be that they expected those would be more welcoming than orientations associated with traditional academic programs. Trainees from *Ancillary Clinical Professions* also chose programs that were significantly higher on Humanistic but lowest in Analytic/Psychodynamic emphasis. It might be that they have encountered humanistic approaches in prior clinical work contexts. *Novice Psych Students* were in programs significantly less in Analytic/Psychodynamic orientation. Again hypothetically, this may reflect the relatively low esteem in which Analytic/Psychodynamic approaches are held in some academic departments. Theoretical orientation is just one factor that determines which programs trainees apply to, and are accepted at—yet this exploratory study shows that therapists’ pathways into the profession can be consequential for trainees choices.

#### Family pathways program correlates

At an earlier – and perhaps deeper – level of influence, trainees from *Nurturing Fami*lies were significantly more often in programs emphasizing Cognitive-Behavioral and deemphasizing Analytic/Psychodynamic and other approaches. By contrast, trainees from *Challenging Families* and *Adversity-marked Families* were significantly more often in Analytic/Psychodynamic programs. This interesting contrast may reflect the appeal of Analytic/Psychodynamic theory, with its characteristic focus on early experience, to trainees trying to make sense of their own problematic past (Table 3), whereas ‘healthy-minded’ trainees from *Nurturing Families* would be drawn to the ‘problem-solving’ conceptual language and focus on the present of Cognitive-Behavioral therapies.

### National correlates

There are clear patterns of national educational pathways: Almost all *Unconventional Novice Candidates* came from Austria, where regulations allow those without academic degrees, or with degrees in other fields, to apply for psychotherapy training. Similarly, a majority of *Ancillary Clinical Professionals*, and a majority of *Advanced Psych Trainees*, lived in Finland, perhaps reflecting incentives and motivation to upgrade their professional status. As the primary educational-occupational pathway, *Novice Psych Students* were found in all five countries, but significantly more than average in Italy and significantly less than average in Finland. Viewing by country, the great majority of trainees in Germany, Italy and – though less emphatically – also the United Kingdom were *Novice Psych Students*, with hardly any *Unconventional Novice Candidates* or *Ancillary Clinical Professionals*. Although these results are unsurprising, because national regulations allow only for certain pathways [8, 25], they do externally validate our educational-occupational cluster solution, clearly mapping differences between nations.

Turning to Childhood Family pathways, a striking contrast appeared between two nations. In Germany, a majority of trainees started from Nurturing Families, whereas less than a third in the UK came from Nurturing Families; in fact, a large plurality of trainees in the comparison countries except the UK came from Nurturing Families. Conversely, a majority of trainees from the UK started from Adversity-marked Families, whereas only a fourth did in Germany. These deviations from base rates were significant. This contrast in Childhood Family pathways between UK and German trainees led us to conduct some post-hoc analyses of further data. It appears that 34% of the UK trainees, in contrast to 10% of German trainees, grew up in large families (4 or more children). Financially, 80% of the German trainees described that they came from economically comfortable families, as compared to 53% in the UK; 47% of trainees in the UK came from families that either had barely sufficient or very marginal material resources as compared to 20% of those in Germany. More than a fourth of the UK trainees said they would be regarded as from a minority group, in contrast to 3% of those in Germany. These post-hoc findings hint at a relatively privileged, mainstream social background for our German trainees (see also [10]). Many trainees in the UK were not so fortunate.

## Limitations

Being the first investigation into pathways to psychotherapy training, no hypothesis could be gathered from previous research, and the approach had to be predominantly exploratory. For the future, more hypothesis testing approaches may be possible.

As most trainees came from European countries, it is not known how relevant our findings may be for other countries. In this context it is informative to look at variables that were not retained in the cluster analysis: the sample is skewed to Christian and non-religious traditions; minority status is quite rare; parental divorce or separation was rather common and covaried with family function and family economic status. Thus the sample is clearly not representative for many countries. Even for the countries studied, data collections were not randomized. Overall, the samples are of unknown representativeness which also means that we do not know how robust the clusters found here are. Moreover, the measurement of the constructs is potentially biased by unknown selection processes leading the trainees to the included programs.

As a contemporary study of adult trainees, none of the historical circumstances of their education and childhood family experiences could be directly observed. Particularly, the trainees’ assessments of their childhood families reflect their current memories and feelings. Thus, it has to be kept in mind that the term “pathway patterns” in this paper does not imply causation but refers to a description of patterns as they are reported by the trainees. Our interpretations must be considered informed speculations and viewed as provisional.

## Future research

A major strength of the study is its large multinational sample with substantial subsamples from different countries. The cluster analytical approach is appropriate at the current “natural history” or observational stage of research as it “botanizes” the trainees’ histories. We hope that the study will open new options for psychotherapy training research, and can envision a number of potential investigations. First would be to collect new trainee samples to see whether the pathway patterns and the correlates found can be replicated. Second would be to extend data collection to countries that have not yet been studied, to explore the generality and limits of our findings. A third, and important area for future research would be to explore the potential effects of pathway patterns to training on other aspects of training programs and trainees’ experiences in them as well their further professional development as practicing psychotherapists, initially and over time. Results from respective research may in the longer run lead to adaptations in training structures and elements [15].

## Supplementary Information

Supplementary Material 1.

## Data Availability

Data are available from the corresponding author (UW) upon reasonable request.

## Footnotes

1 In order to do justice to the variability of how psychotherapy and psychotherapists are defined internationally we have used the self-identification of training institutes as being psychotherapy training centers.

2 Thus, the concept of pathways used in this paper is more specifically and quantitatively empirical than the broader sense used when introduced in Farber et al.’s paper [7].

3 TBIF-structured response items are derived from the DPCCQ [18]. TBIF text responses to open answers were coded by two rater pairs (AH and DO; JP and UW). Finnish-language responses were translated and cross-checked by EH. Data from India were categorized by PB.
