# Supplementary material for: Pathways to professional training: a multinational study of psychotherapy trainees
Source: BMC Psychol. 2026 Aug 24;14:1246. doi: 10.1186/s40359-026-05133-w (PMC13501768; doi:10.1186/s40359-026-05133-w)
Supplement: Supplementary file 1 [file 40359_2026_5133_MOESM1_ESM.docx]

**Supplement 1/Online-Material**

**Table A1: Relation of Educational-Occupational and Childhood Family Pathways**

| **Educational-\Occupational**  **Pathways** | | **Family Pathways** | | | | |  |
| --- | --- | --- | --- | --- | --- | --- | --- |
|  |  | **Nurturing Families** | | **Challenging Families** | | **Adversity-marked Families** | Total |
| **Novice**  **Psychology/iatry**  **Students** | *n* | 302 | | 143 | | 189 | 634 |
|  | % | 58.3% | | 50.5% | | 62.8% | 57.5% |
|  | AR**^1^** | .5 | | -2.8 | | 2.2 |  |
| **Advanced**  **Psychology/iatry**  **Trainees** | *n* | 101 | | 72 | | 44 | 217 |
|  | % | 19.5% | | 25.4% | | 14.6% | 19.7% |
|  | AR**^1^** | -.2 | | 2.8 | | -2.6 |  |
| **Ancillary**  **Profession**  **Trainees** | *n* | 55 | | 37 | | 40 | 132 |
|  | % | 10.6% | | 13.1% | | 13.3% | 12.0% |
|  | AR**^1^** | -1.3 | | .7 | | .8 |  |
| **Unconventional**  **Novice**  **Candidates** | *n* | 60 | | 31 | | 28 | 119 |
|  | % | 11.6% | | 11.0% | | 9.3% | 10.8% |
|  | AR**^1^** | .8 | | .1 | | -1.0 |  |
| Total | *n* | 518 | | 283 | | 301 | 1102 |
| Pearson Chi-Square = 15.05 | | | df = 6 | | *p* = .02 | | |
| 1 - AR =Adjusted standardized residual. | | | | | | | |

**Supplement 2/Online-Material**

**Supplement 2/ Ethics committee approvals and informed consent**

Ethics committee approvals for the SPRISTAD study

As SPRISTAD is an international study cooperating training sites applied to their local ethics committees for approval to the study. After that they recruited participants from their training center by providing standardized information about the study. Participants signed the informed consent, provided their e-mail address and the data collection center at Witten/Herdecke university sent them a link to the forms. Trainees could drop out from the study any time without explanation or negative consequences. Training centers got no information about who participated in the study. Once more than 10 trainees participated from a training center the respective center could get summary information about trainees‘ responses without identifying information about particular respondents.

Below you find exemplary SPRISTAD-study approvals of the ethics committees (in accordance with the Helsinki Declaration and respective follow-ups):

- University of Padova (Italy), applied by Ass. Prof. Irene Messina: Approval by the ethics committee from 2015/10/08, reference number: 2860BD1DF571631FA7D115B2ED2D0C8
- University of Helsinki (Finland), applied by Prof. Erkki Heinonen: Approval by Ethical review board in humanities and social and behavioral sciences 8/2015 (no reference number)
- Regional Committee for Medical and Health Research Ethics, Oslo (Norway), applied by Prof. Dr. Hanne Stromme (University of Oslo): Approval by the Ethics Committee from University of Oslo from 2015/11/10 (reference number: IRB00001870REK Sør-Øst C)
- Sigmund-Freud-University, Vienna (Austria), applied by Prof. Dr. Omar Gelo: Approval by the Ethics Committee from 2015/11/17 (no reference number)
- University of Heidelberg (Germany), Ethikkommission der Fakultät für Verhaltens- und Empirische Kulturwissenschaften, applied by Dr. Johannes Mander: Approval of the Ethics Committee from 2016/12/08 (no reference number)
- Medical University of Vienna (Austria), applied by Prof. Dr. Henriette Löffler-Stastka: Approval by the Ethics Committee from 2016/01/16 (Reference number: EK-NR: 2171/2015)
- Comite di Bioethica, Buenos Aires (Argentina; <https://irbioetica.com>), applied by Prof. Beatriz G6mez Fundaci6n Aigle: Approval by the ethics committee from 2017/05/29 (no reference number)
- University of Sherbrooke (Canada) Comité d’éthique de la recherche – Lettres et sciences humaines,, applied by Prof. Laverdière: Approval by the ethics committee from 2017/12/01 (reference number: N/Réf. 2017-1551/Laverdière)
- University Witten/Herdecke IRB (Germany), applied by Prof. Dr. Ulrike Willutzki: Approval by the ethics committee from 2017/11/07
- University of Berne IRB (Switzerland), applied by Prof. Dr. Franz Caspar: Approval by the ethics committee in November 2017 (no reference number)
- Northwestern University Chicago IRB, applied by Dr. Ryan Earl (Center for Applied Psychological & Family Studies, Chicago, USA): Approval by the ethics committee from 2018/10/11 (reference number: STU00207851)
